# Prenatal ultrasound and the risk of childhood brain tumour and its subtypes

**DOI:** 10.1038/sj.bjc.6604284

**Published:** 2008-03-18

**Authors:** K Stålberg, B Haglund, O Axelsson, S Cnattingius, S Pfeifer, H Kieler

**Affiliations:** 1 Department of Women's and Children's Health, Obstetrics and Gynecology, Uppsala University, Uppsala 75185, Sweden; 2 Centre for Epidemiology, National Board of Health and Welfare, Stockholm 10630, Sweden; 3 Department of Medical Epidemiology and Biostatistics, Karolinska Institutet, Box 281, Stockholm 17177, Sweden; 4 Department of Genetics and Pathology, Rudbeck Laboratory, Uppsala University, Uppsala 75185, Sweden; 5 Centre for Pharmacoepidemiology, Karolinska Institutet, Stockholm 17176, Sweden

**Keywords:** brain tumour, childhood, ultrasound, epidemiology

## Abstract

We carried out a nationwide case–control study of childhood brain tumours in Sweden (*n*=512) by histological subtype in relation to prenatal ultrasound, extracting data from antenatal records and the Medical Birth Register. We found no increased risk for brain tumour after ultrasound exposure, either for all tumours or for any subgroup.

Since prenatal ultrasound scanning was introduced more than 40 years ago (Donald *et al*, 1958), ultrasound machines have multiplied their acoustic output several fold (Henderson *et al*, 1995), scans are more frequent and Doppler ultrasound is used. During this same period, the incidence of childhood brain tumours (CBT) has increased (Hjalmars *et al*, 1999; NCI, 2005), although whether this reflects a true increase or merely an improved diagnosis or reporting is not known (Smith *et al*, 1998). Thus, it is important to determine if environmental factors such as prenatal ultrasound could have contributed to the observed increase.

Previous studies on prenatal ultrasound exposure and childhood cancer have failed to show any clear association (Cartwright *et al*, 1984; Kinnier Wilson and Waterhouse, 1984; Bunin *et al*, 1994; Shu *et al*, 1994; Sorahan *et al*, 1995; Naumburg *et al*, 2000); only a few studies have evaluated CBT separately (Cartwright *et al*, 1984; Bunin *et al*, 1994; Shu *et al*, 1994) and only one published study has evaluated prenatal ultrasound according to CBT subtypes (Bunin *et al*, 1994). These studies were based on retrospectively collected data and recall bias cannot be ruled out.

In the present nationwide population-based case–control study, we used prospectively recorded exposure data to study the associations between prenatal ultrasound exposure and CBT subtypes.

## MATERIALS AND METHODS

In the nationwide Swedish Cancer Register, we identified as cases 601 children born between 1975 and 1984 and with a diagnosis of brain tumour (ICD-7 code 193) before the age of 15. The same number of controls were randomly selected from the Medical Birth Register and frequency matched to cases by gender and year of birth. With this sample and assuming a power of 80%, a two-sided 5% significance level and an ultrasound exposure frequency of 50%, we estimated that we should be able to detect an odds ratio (OR) of at least 1.4 for all CBT. For 62 out of the 601 cases, the information on personal identification numbers or hospital of birth was incomplete or missing, which made it impossible to identify their antenatal records. Out of the remaining 539 cases, we retrieved antenatal records for 512 (95%), and for the 539 controls, 524 antenatal records (97%).

Information on mother's reproductive history and obstetric parameters was retrieved from the Medical Birth Register. From the antenatal records, we extracted information on ultrasound exposure, including gestational age at exposure and number of examinations. All data collections were blind to case/control status. Individual record linkage was made possible by the personal identification number assigned to each Swedish resident at birth.

Associations were evaluated for all types of CBT combined and by the following subtypes: low-grade astrocytoma, high-grade astrocytoma, primitive neuroectodermal tumour (PNET), ependymoma, germ-cell tumour or other rare and incompletely specified tumours.

We used logistic regression to evaluate the association between prenatal exposure to ultrasound and the incidence of CBT. Estimates of ORs and 95% confidence intervals (CIs) were calculated. *A priori*, we identified potential confounding factors that could interfere with both ultrasound exposure and outcome (CBT), and for which it was possible to retain information from either the registers or the antenatal records. The following confounders were included in the adjusted analyses: maternal age at birth, parity, multiple births, mother's country of birth (Nordic (Sweden, Norway, Denmark, Finland and Iceland) or non-Nordic country), mother's smoking habits, hypertension, mode of delivery, breech position, gestational age at birth, birth weight, head circumference at birth and the level of hospital where born. Statistical analyses were conducted with the SAS 9.1 software package. A detailed description of population and study methods has been published elsewhere (Stalberg *et al*, 2007).

This study was approved by the Ethics Committees at Karolinska Institutet and Uppsala University.

## RESULTS

In children with CBT, 50.4% were boys and 49.6% girls. Among children with CBT, it was more common to be the first-born child (*P*=0.01), and to be born at a primary- or secondary-level hospital (*P*=0.04), than for controls. No other significant differences between cases and controls were seen in maternal and neonatal characteristics (Stalberg *et al*, 2007). The median age for diagnosis for all CBT was 8 years. For the subtypes, the median ages were as follows: ependymoma, 4 years; PNET, 6 years; low- and high-grade astrocytoma, 8 and 9 years, respectively; and germ-cell tumours, 9 years.

The overall exposure rate for ultrasound was 44.1% (*n*=226) for case mothers and 45.7% (*n*=240) for control mothers. All ultrasound examinations were performed abdominally and none involved Doppler ultrasound. In Table 1, the distributions of the CBT subtypes and ORs according to prenatal ultrasound exposure are presented. Being exposed to prenatal ultrasound was not associated with an increased overall risk of brain tumours compared with being unexposed (adjusted OR 1.00, 95% CI: 0.77–1.29). When stratifying according to histological subgroups, no increased risks were observed for low- and high-grade astrocytomas or for PNET. For ependymomas and germ-cell tumours, cases were too few (44 and 17, respectively) to perform multivariate analyses with adjustments for possible confounders. However, there were no increased risks seen in the crude risk estimates (presented in Table 1).

In Table 2, crude and adjusted ORs for all CBT by trimester of ultrasound exposure are shown, including exposure exclusively in one trimester and for combinations with other trimesters. No specific trimester of ultrasound exposure or any combination of trimesters was associated with a significantly increased risk of CBT. The highest OR was observed for exposure in the second trimester, together with at least one exposure in another trimester (adjusted OR 1.27, 95% CI: 0.85–1.90). Being exposed to two or more ultrasound examinations was not associated with any significantly increased risk of CBT, compared with being unexposed (crude OR 0.97, 95% CI: 0.71–01.32; adjusted OR 1.09, 95% CI: 0.78–1.52).

## DISCUSSION

This is the first study on prenatal ultrasound exposure and subsequent risk of CBT subtypes using prospectively recorded exposure information. In agreement with previous studies, we found no overall increased risk for any separate subtype. The trimester of exposure and number of ultrasound examinations had no impact on risks. The finding of no increased risk for PNET, which is the only subtype of CBT arising from neurones, is in contrast to the moderately increased risks of PNET by prenatal X-ray exposure recently reported from the same cohort (Stalberg *et al*, 2007).

In most case–control studies of prenatal ultrasound exposure and CBT risk, the number of cases was small, ranging from 77 to 107 (Cartwright *et al*, 1984; Shu *et al*, 1994). Consequently, none of these studies had sufficient statistical power to study a moderate association between prenatal ultrasound and CBT. In a study of 321 cases based on retrospective interviews, no increased risks were seen for astrocytomas and PNET (Bunin *et al*, 1994). Our study has the advantage of using exposure data prospectively recorded during pregnancy, which precludes recall bias. Other strengths include its population-based design, the blinded data collection and the few missed cases, which minimize selection bias.

We had the opportunity to control for a number of possible confounders; these had only minor effects on the results. However, confounding by indication can be important. In Sweden, most foetuses are scanned on a routine basis in the second trimester and further scans are generally performed by indication. The highest OR was seen for children scanned in the second and at least one more trimester (adjusted OR 1.27, 95% CI: 0.85–1.90). Although not statistically significant, this slightly increased risk may indicate that children followed-up with further scans had some confounding factor unknown to us and therefore not adjusted for.

Ultrasound can damage the biological tissue by heating, cavitation or streaming. Whether any of these mechanisms is carcinogenic is not known, but experimental studies on tissue cultures have shown that ultrasound with intensities used for prenatal scanning can damage cell membranes (Dinno *et al*, 1989). In some of the first reports on potential hazards by ultrasound, chromosomal damages, including sister chromatid exchanges, were described (Liebeskind *et al*, 1979) but could not be confirmed by later studies.

One of the main potential biological effects of prenatal ultrasound is heating. Bone has the highest absorption coefficient for heat, and as the CNS tissues are encased in the skull or vertebrae, the CNS can be subjected to heating by conduction (Barnett, 1998). The increase in temperature by Doppler ultrasound is higher than for B-mode ultrasound only (Barnett, 2001). The individuals in this study had been exposed to imaging ultrasound and not to Doppler ultrasound.

No carcinogenic effect of prenatal ultrasound exposure was found in this or previous studies (Cartwright *et al*, 1984; Kinnier Wilson and Waterhouse, 1984; Bunin *et al*, 1994; Shu *et al*, 1994; Sorahan *et al*, 1995; Naumburg *et al*, 2000). Although reassuring, these studies assessed ultrasound exposure in the 1970s and 1980s, when intensity output levels for ultrasound machines averaged around 20 mW cm^−2^ spatial peak temporal average intensity (Duck and Martin, 1991; Kieler *et al*, 1998). In 1993, the United States Food and Drug Administration set an overall limit of 720 mW cm^−2^ for any type of ultrasound examination (FDA, 1993). The suspicion of much higher energy exposures nowadays compared with 20 or 30 years ago in combination with more frequent scans and the use of Doppler ultrasound means that possible adverse effects of prenatal ultrasound scanning need to be monitored in the future.

## Figures and Tables

**Table 1:** Odds ratios and 95% CIs for all childhood brain tumours combined and by subtype in relation to prenatal ultrasound

		**Crude**		**Adjusted^a^**	
		**OR**	**95% CI**	**OR**	**95% CI**
	*N* b				
All brain tumours^c^	503	0.94	0.73–1.20	1.00	0.77–1.29
Astrocytoma (low-grade)	190	0.96	0.68–1.34	1.02	0.72–1.44
Astrocytoma (high-grade)	60	0.86	0.50–1.48	1.10	0.62–1.96
PNET	104	0.85	0.55–1.30	0.85	0.54–1.35
Ependymoma^d^	42	0.67	0.35–1.29	—	—
Germ-cell tumours^d^	17	1.07	0.41–2.82	—	—

Abbreviations: CI=confidence interval; OR=odds ratio; PNET=primitive neuroectodermal tumour.

a Adjusted for maternal age, parity, multiple birth, mother born in a Nordic country, gestational age at birth, mode of delivery, breech position, birth weight, head circumference at birth, level of hospital, hypertension during pregnancy and maternal smoking.

b Nine subjects were excluded because information on variables adjusted for was missing.

c Including the subtypes in the table and other miscellaneous tumours (*n*=90).

d Multivariate analyses could not be performed because of the low number of cases.

**Table 2:** Odds ratios and 95% CIs for all childhood brain tumours in relation to trimester of ultrasound exposure

				**Crude**		**Adjusted^a^**	
		**Cases**	**Controls**	**OR**	**95% CI**	**OR**	**95% CI**
	Unexposed^b^	283	280	1.00		1.00	
First trimester (weeks 2–14)	Only first	12	17	0.70	0.33–1.49	0.75	0.34–1.63
	First and other^c^	24	28	0.85	0.48–1.50	0.96	0.53–1.73
Second trimester (weeks 15–28)	Only second	64	62	1.02	0.69–1.50	1.05	0.70–1.56
	Second and other^c^	70	62	1.12	0.76–1.63	1.27	0.85–1.90
Third trimester (weeks 29–45)	Only third	53	69	0.76	0.51–1.13	0.82	0.54–1.24
	Third and other^c^	65	67	0.96	0.66–1.40	1.10	0.74–1.65

Abbreviations: CI=confidence interval; OR=odds ratio.

Only subjects with information on all variables adjusted for are included. Thirty subjects with uncertain trimester of ultrasound exposure were excluded.

a Adjusted for maternal age, parity, multiple birth, mother born in a Nordic country, gestational age at birth, mode of delivery, breech position, birth weight, head circumference at birth, level of hospital, hypertension during pregnancy and maternal smoking.

b Reference group.

c Individuals were also exposed to ultrasound in at least one of the other trimester. Consequently, one individual can be included in more than one of the ‘other’ row.
